# Prevalence and Associated Factors of *Blastocystis* sp. Infection in Patients with Gastrointestinal Symptoms in Spain: A Case-Control Study

**DOI:** 10.3390/tropicalmed7090226

**Published:** 2022-09-03

**Authors:** Cristina Matovelle, María Teresa Tejedor, Luis Vicente Monteagudo, Antonio Beltrán, Joaquín Quílez

**Affiliations:** 1 Faculty of Medicine, University of Zaragoza, 50009 Zaragoza, Spain; 2 Environmental Sciences Institute (IUCA), University of Zaragoza, 50009 Zaragoza, Spain; 3 Department of Anatomy, Embryology and Animal Genetics, Faculty of Veterinary Sciences, University of Zaragoza, 50013 Zaragoza, Spain; 4 Aragon Institute of Health Sciences (IACS), CIBERCV, 50009 Zaragoza, Spain; 5 AgriFood Institute of Aragon (IA2), 50013 Zaragoza, Spain; 6 Service of Microbiology and Parasitology, Hospital Clínico Universitario Lozano Blesa, 50009 Zaragoza, Spain; 7 Department of Animal Pathology, Faculty of Veterinary Sciences, University of Zaragoza, 50013 Zaragoza, Spain

**Keywords:** *Blastocystis* sp., gastrointestinal symptoms, intestinal protozoa, relative eosinophilia, Spain

## Abstract

*Blastocystis* sp. is known to be the most prevalent parasite in fecal samples of humans worldwide. In the present report, a case–control study (1:9.89 (≈10)) was performed, by analyzing data from 3682 patients who attended a public hospital in the northern area of Spain showing gastrointestinal symptoms. Diagnosis was performed in human fecal samples by means of optical microscopy. The prevalence of *Blastocystis* sp. in patients with gastrointestinal symptoms was 9.18% (338/3682). Most of the *Blastocystis* sp.-infected patients tested negative for protozoa and helminths, and were underweight and foreign-born (26.4%), mainly from Africa and Central/South America. Gastrointestinal symptoms, such as abdominal pain, anorexia, halitosis, plus relative eosinophilia, as well as co-infections with pathogenic bacteria were associated with *Blastocystis* sp. infection. Both type 2 diabetes and treatment with immunosuppressive medicines at the time of *Blastocystis* sp. detection were associated with a higher proportion of infected patients. This is the first case–control study of *Blastocystis* sp. in humans in northern Spain and may contribute to surveillance and intervention strategies by public health authorities.

## 1. Introduction

*Blastocystis* sp. is the most prevalent enteric protist reported in human fecal samples [1,2]. Over one billion people are estimated to be infected with *Blastocystis* sp. worldwide, especially in developing countries, where infection frequencies exceeding 50% are commonly reported, and even up to 100% in some tropical countries [3,4,5]. Conversely, the prevalence is significantly lower in developed countries (10–15%) [6]. The variations in prevalence between studies could be attributed to different factors, including the use of different diagnostic approaches in clinical microbiology laboratories, with many largely underestimating the prevalence in the context of enteric parasite diagnosis [7,8]. Nevertheless, it is well known that the high prevalence of this protist in developing countries is related to socioeconomic factors that lead to poor sanitation and higher potential sources of infection, including human-to-human, zoonotic, and waterborne transmission [9].

Although this parasite was discovered more than a century ago, its pathogenic mechanisms still remain under discussion. Several studies support the pathogenic potential of *Blastocystis* sp. in humans by reporting gastrointestinal symptoms, such as abdominal pain, diarrhea or vomiting, as well as a diverse spectrum of cutaneous symptoms, mainly urticaria, in the absence of any other cause of sickness identified in patients [10,11]. In contrast, some other studies did not report conclusive evidence for the pathogenic role of *Blastocystis* sp. and consider this protist a common microorganism of healthy intestinal microbiota [12,13]. In the last decade, an increasing number of studies have explored the genetic diversity of *Blastocystis* sp. in humans. A total of 33 different subtypes have been reported to date in humans and animals based on variations in the small subunit ribosomal RNA (SSU-rDNA) gene [14]. However, not all strains of a specific subtype are clinically relevant and a possible correlation between the different subtypes and their pathogenic potential is still strongly debated [15,16,17].

Multiple risk factors have been reported for *Blastocystis* sp. human infections, including poor hygiene conditions, not washing hands after using toilets, drinking non-tap water and contact with animals [18,19]. The prevalence of the parasite was also correlated with a low socioeconomic status, low education and poor health conditions [20]. Age, nutritional status and some clinical conditions (anemia, irritable bowel syndrome) have also been linked to *Blastocystis* sp. infections [19,21]. In European countries, travelling abroad or infections with other enteric parasites significantly increase the risk of having a positive result for *Blastocystis* sp [17,22,23].

In Spain, studies on the prevalence and risk factors of *Blastocystis* sp. infection in humans are limited and mostly focused on specific groups of the population. Studies in asymptomatic and symptomatic children have revealed prevalence rates of 5.3–19.4% using microscopy and/or PCR-based techniques, and up to 27.8% of adult patients tested positive for *Blastocystis* sp. using both diagnostic methods [24,25,26,27]. Adult age, working with the public, being African and travelling to other countries have been reported to significantly increase the risk of having a positive diagnosis of *Blastocystis* sp., while good hygiene practices, such as hand or vegetable washing, have been recommended to minimize the risk [23,26,28,29]. Few studies in Spain have analyzed the relationships of this parasite with the presence of digestive symptoms, which was not found as a significant factor in patients older than 18 years [23]. The current case–control study was aimed to analyze the prevalence of *Blastocystis* sp. in patients of different age groups with gastrointestinal manifestations in northern Spain. In addition, co-infection of *Blastocystis* sp. and other species of pathogenic bacteria, helminths and protozoa were analyzed. Multiple variables, including anthropometric, socio-demographic and clinical data, were analyzed as potential associated factors with *Blastocystis* sp. infection.

## 2. Materials and Methods

### 2.1. Ethics Approval Statement

The present study was performed following the guidelines of the Declaration of Helsinki in 1975, revised in 2013. All procedures performed in this study were approved by the Ethics Committee of Aragón (ref 18/081) before undertaking this research to confirm that the study meets national and international guidelines. A signed informed consent was obtained from every participating patient before they participated in the study, and patients were completely anonymized by the researchers. All the authors ensure that this study is HIPAA (Health Insurance Portability and Accountability Act, 1996) compliant. The researchers followed every mandatory (health and safety) procedure.

### 2.2. Study Population

The study was conducted using fecal specimens from patients with gastrointestinal illness submitted for microbiological and parasitological diagnosis to the Microbiology and Parasitology Department of the Hospital Clínico Universitario Lozano Blesa (Zaragoza, Spain) in 2018. This is a public hospital that cares for a combined urban and rural population of more than 274,000 citizens in Zaragoza. Increasing immigrations rates have been reported in this sanitary area in the last few years. Individuals born in foreign countries, mainly from tropical and subtropical regions, and those that have access to the Spanish universal health coverage were also included in this study. Overall, 6087 specimens from 3682 patients were analyzed for both stool culture and parasitological diagnosis from 1 January 2018 to 31 December 2018. Patients were defined as cases if they tested positive for *Blastocystis* by microscopy and patients with negative results were defined as controls. The mean age of the individuals selected as cases and controls was 30.18 ± 1.294 years (range: 1 to 100 years).

Demographic, anthropometric and medical variables from all patients were obtained using (1) the Modulab^©^ Werfen Gold version 2.0 laboratory computer program (Werfen Spain, L’Hospitalet de Llobregat, Barcelona. Spain), and (2) the Intranet electronic program (website of the Aragón Healthcare Service). The categorical variables analyzed for association with *Blastocystis* sp. infection were as follows:

- Demographic origin: Spain, rest of Europe, Africa, America and Asia;
- Age group: ≤16 years and >16 years;
- Sex: male and female;
- Categorized body mass index (BMI): underweight (<18.4 Kg/m^2^), normal weight (18.5–24.9 Kg/m^2^), overweight (25–29.9 Kg/m^2^) and obesity 30–>40 Kg/m^2^);
- Immunosuppressive treatment: chemotherapy, monoclonal antibodies, antiretroviral drugs, nucleoside analogs, corticosteroids;
- Gastrointestinal symptoms: diarrhea, abdominal pain, nausea, vomiting, constipation, anorexia, fever, aerophagia, halitosis, urticaria, anal itching and dyspepsia;
- Co-morbidities: celiac disease (CD), type 2 diabetes, IBS (irritable bowel syndrome) and cancer;
- Laboratory parameters: glucose (normal range: 82–115 mg/dL), glycosylated hemoglobin (normal range: 4.6–5.7%), hemoglobin (normal range: 12–15.3 g/dL) and relative eosinophilia (percentage of eosinophils on total leukocyte count, normal range: 2–10%);
- Co-infection with pathogenic bacteria or other enteric protozoa or helminths.
- Age and BMI were also analyzed as continuous variables.

It should be noted that some of these variables were not available for all positive patients.

### 2.3. Stool Examination

The standard procedure for *Blastocystis* sp. diagnosis in the Clinical Microbiology and Parasitology laboratory in the Hospital Clínico Universitario Lozano Blesa was applied. Briefly, fecal specimens were concentrated by the SAF (sodium acetate-acetic acid -formalin) sedimentation method and examined by light microscopy. Three stool wet-mount preparations for each fecal specimen were evaluated for the presence of *Blastocystis* sp., as well as protozoa and helminths. The entire preparations from left to right and from top to bottom were examined with 10× and 40× optical objective lenses. Diagnosis of *Blastocystis* sp. was based on the morphology of the parasites observed in the preparation [1,30]. The Ziehl–Neelsen modified technique was used to screen for *Cryptosporidium* oocysts. Those patients that submitted repeated fecal samples on days 1-3-5 were considered positive for *Blastocystis* sp. if any of the samples were positive.

Furthermore, optical microscopic examination detected several microorganisms, such as Protozoa (*Cryptosporidium* spp., *Dientamoeba* spp., *Encephalitozoon* *hellem*, *Entamoeba* spp., *Giardia lamblia* and *Isospora belli*), and Helminths (*Ascaris lumbricoides*, *Enterobius vermicularis*, *Hymenolepis nana*, *Schistosoma intercalatum*, *Strongyloides stercolaris*, *Taenia saginata* and *Trichuris trichiura*) [30].

For the microbiological stool analysis, samples were cultured using Hektoen agar (Biomerieux^®^, Hazelwood, MO, USA), Mac Conkey (Biomerieux^®^, Hazelwood, MO, USA), CCDA agar (Biomerieux^®^, Hazelwood, MO, USA), and selenite broth with reseeding on Hektoen after incubation for 16 h. In patients with blood in the samples, seeding in Mac Conkey/sorbitol agar was added. Incubations were carried out at 37 °C, except for CCDA agar, which was incubated at 45 °C in anaerobiosis [31]. MALDI-TOF MS was used to identify bacteria at the species level (*Campylobacter* sp., *Arcobacter butzleri*, *Aeromonas* sp., *Salmonella* sp., *Shiguella* sp., *Yersinia* sp., *Escherichia coli*) [32].

### 2.4. Data Analysis

Statistical analyses were carried out using the IBM SPSS Statistics 26.0 software package (IBM Corp., Armonk, NY, USA). For categorical variables, proportions and percentages were calculated; Pearson’s chi-squared (with continuity correction for 2 × 2 tables) or, alternatively, Fisher’s exact test (expected number < 5) was used for comparisons between the case and control groups. For multiple comparisons (more than two categories), Bonferroni correction was used. Age (years), BMI (kg/m^2^) and blood parameters (concentrations of glucose, glycosylated hemoglobin and hemoglobin and relative eosinophilia) were submitted to the Shapiro–Wilks test to assess their normal distribution. Age was summarized by mean and SE, while the median and interquartile range (IQR) were used to summarize the rest of quantitative variables. T-test was used for comparing ages between the cases and controls, while the Mann–Whitney U test was applied for comparisons involving the rest of the quantitative variants. *p* values < 0.05 were considered as statistically significant.

Twenty-one variables are considered in the study of co-infections (20 variables for pathogenic bacteria, helminths and protozoa, plus 1 variable for *Blastocystis*). They were categorical variables, codified as 0 and 1 (absence or presence of a particular pathogen, respectively). Principal component analysis (PCA) was used to reduce a large set of variables for co-infections into a smaller set of variables (principal components), accounting for most of the variance in the original variables; PCA provided a basis of statistical significance to the presence of two or more microorganisms in the same individual. Microorganisms present only in few individuals or those that lacked a relationship with each other were eliminated from the study; the co-infection in these cases could be merely a fortuitous event. The KMO index (Kaiser–Meyer–Olkin index) measures the linear relationship between the variables; the KMO index for a particular variable must be >0.5 for that variable to be considered for the PCA [33]. The eigenvalue-one criterion [34] and the scree plot [35] were used in order to decide the number of components to be retained. Orthogonal rotation (varimax) was chosen. Since we are interested in grouping individuals on the basis of co-infections, a cluster analysis was applied on the retained principal components. Initially, a hierarchical agglomerative procedure was used, creating the clusters by means of Ward’s method to define the number of clusters by the elbow rule. Then, the k-means procedure was used to form the clusters [36]. These clusters are groups of individuals that share similar characteristics about co-infections (or absence of co-infections); hence, the parasitic characteristics of these clusters can be interpreted as the most interesting co-infections (or absence of co-infections), based on their frequencies and the significant association of the involved parasites.

Those variables that showed significant differences between the cases and controls were chosen for logistic regression analysis. The association of these variables (independent variables) with case/control status (dependent dichotomous variable; value labels: case = 1, control = 0) was ascertained by binomial logistic regression. The model was adjusted using a stepwise procedure (method: forward; Wald test); the significance levels for the variables to enter and to be removed were *p* ≤ 0.05 and *p* ≥ 0.10, respectively [37]. Nagelkerke R^2^ was used to estimate how much variation in the dependent variable can be explained by the model. The model’s ability to discriminate between cases and controls was assessed by its accuracy parameters, which were as follows: sensitivity (Sn: true positive rate), specificity (Sp: true negative rate), positive and negative predictive values (PPV and NPV: proportions of positive and negative results in diagnostic tests that are true positive and true negative results, respectively) [38]. The overall measure of discrimination was represented by the area under the receiver operating characteristics (ROC) curve [39]. The 95% confidence interval (95% CI) was estimated for both accuracy parameters and area under the ROC curve.

## 3. Results

**Figure 1:**
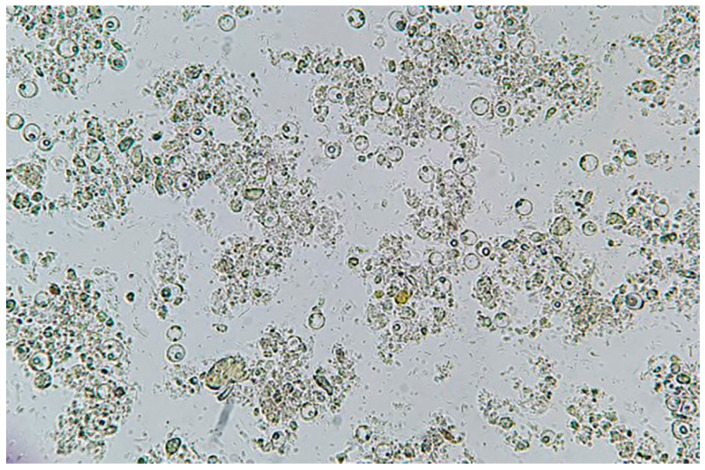
Microscope field image (100× *g* magnification) obtained in a sample from an infected *Blastocystis* patient with high parasite load.

Fecal specimens from 338 of 3682 patients (9.18%) tested positive for *Blastocystis* sp. infection and were included as cases. Figure 1 shows a microscope field obtained in a *Blastocystis* positive sample.

The control group consisted of 3344 patients negative for this parasite. The case–control ratio group was 1: 9.89 (≈10). Table 1 shows the distribution of the demographic/anthropometric parameters and treatment with immunosuppressive drugs in positive and negative *Blastocystis* sp.-infected patients. Most of the variables showed statistically significant differences between the cases and controls. In relation to the geographical origin, the percentage of patients from Spain was significantly lower in the cases than in the controls, while the situation was reversed in the patients from other countries, with significant differences in those from Africa, America (Central and South America, plus one *Blastocystis* negative patient from USA). When age was categorized based on a 16 years cut-off (child vs. adult), no significant difference was detected between cases and controls; in contrast, the cases were significantly younger than controls when age was considered as a quantitative variable. Underweight individuals were significantly more common in the cases than in controls; in contrast, both normal weight and overweight persons were significantly more frequent in the controls. The BMI as a quantitative variable also differed significantly between cases and controls, with lower values in the cases. Immunosuppressive treatment with chemotherapy, monoclonal antibodies or antiretroviral drugs were significantly more common in the cases than in controls, but treatment with corticosteroids was more frequent in the controls. Sex and proportion of patients undergoing treatment with nucleoside analogs did not differ between cases and controls.

The clinical symptoms, underlying diseases, co-infections with pathogenic bacteria or other enteric parasites and laboratory test results in the cases and controls are summarized in Table 2. Most symptoms were significantly more common in the cases than in controls, including abdominal pain, nausea, anorexia, aerophagia, halitosis, urticaria, anal itching and dyspepsia. In contrast, the presence of diarrhea, and fever was more common in the controls than in cases, with statistically significant differences in the presence of fever. No significant differences were detected between the groups for the co-morbidities, except for type 2 diabetes, which was more frequent in the cases, while irritable bowel syndrome (IBS) was significantly more common in negative patients. There was no difference in biochemical parameters between *Blastocystis* sp.-infected and uninfected patients. However, highly significant differences were found for relative eosinophilia, showing greater values in the cases.

Several variables related to the absence/presence of pathogens were not included in the PCA, whose results are described in Table 3. Namely, a very low number of individuals (<2) showed presence of *Arcobacter butzleri*, *Shigella*, *Schistosoma intercalatum*, *Taenia saginata* and *Encephalitozoon hellem*; therefore, these variables were not considered for PCA. Several variables had a KMO index <0.5, breaching the basic assumption for PCA (*Aeromonas* sp., *Campylobacter* sp., *E. coli*, *Salmonella* sp., *Yersinia* sp., *Cryptosporidium* sp., *Hymenolepis nana* and *Trichuris trichiura*). Hence, only *Isospora belli*, *Strongyloides stercoralis*, *Giardia lamblia*, *Dientamoeba, Entamoeba* sp., *Ascaris lumbricoides*, *Enterobius vermicularis* and *Blastocystis* sp. were considered for PCA. Based on the criteria explained in the statistical methodology, three principal components were retained. The cumulative percentage of variance explained by them was 46.996%.

Table 3 shows the rotated component matrix (the key output of PCA); it shows the estimates of the correlation coefficients between each of the original variables and the estimated components. Hence, PCA summarizes the information about co-infection from the original 21 variables in only 3 variables (three principal components; 1, 2 and 3), including the original variables shown in Table 3. The coefficients below 0.3 (absolute value) have been suppressed for easier interpretation. Positive loadings indicate that a variable and a principal component are positively correlated; an increase in one results in an increase in the other. Negative loadings indicate a negative correlation. Large (either positive or negative) loadings indicate that a variable has a strong effect on that principal component. Principal component 1 loads very strongly on *Isospora belli*, *Strongyloides stercoralis* and *Giardia lamblia* but less strongly on *Dientamoeba* sp.; this principal component mainly summarizes the information about the presence of these four agents. Similarly, principal component 3 loads very strongly on *Enterobius vermicularis* and less strongly on *Blastocystis* sp. Finally, principal component 2 loads strongly on *Entamoeba spp* and *Ascaris lumbricoides*, but lightly on *Blastocystis* sp. and *Strongyloides stercoralis* (negative load); this principal component summarizes the information about the presence of *Entamoeba spp* and *Ascaris lumbricoides*, and *Blastocystis* and the absence of *Strongyloides stercoralis*.

Once the original variables set was reduced by PCA, cluster grouping based on the three retained principal components must be applied in order to study co-infections. These clusters are groups of individuals that share similar characteristics, as it refers to the variables included in the three principal components; hence, this provides a simplified view of the most relevant co-infections in the considered sample of individuals. Table 4 shows the result of the clustering based on the three retained principal components. The first cluster included most of the individuals that were negative for *Blastocystis* sp. (3277/3344 = 98%), while the sixth cluster contained the majority of the *Blastocystis* sp. positive patients (301/336 = 89.50%), with no presence of other parasites in either cluster. Hence, most of the studied individuals, either cases or controls, did not show any co-infection. Each cluster of individuals concerned a particular microorganism, since most of the individuals that showed this microorganism are included. Cluster 2 concerned *Giardia lamblia* (45/52 individuals infected with *Giardia* = 86.53%) and included only 9 of the 336 patients infected with *Blastocystis* sp. (2.68%). Cluster 3 included all patients infected with *Enterobius vermicularis* (n: 16) and included eight patients positive for *Blastocystis* sp. (2.38%). Cluster 4 contained all patients infected with *Entamoeba* sp. (n: 19) and included twelve patients positive for *Blastocystis* sp. (3.57%). Finally, all patients infected with *S. stercoralis* were allocated to Cluster 5 (n: 6), which also included six *Blastocystis* sp.-infected patients (1.78%). The Table 4 shows the results obtained for all the kinds of co-infections. Since this study focuses on *Blastocystis*, Table 5 provides a more accurate description of co-infections, including more than one microorganism, in addition to *Blastocystis*.

Table 6 shows the final model for binomial logistic regression; it was statistically significant (χ^2^ (11) = 1955.696; *p* < 0.001) and explained 94.6% of the total variance (Nagelkerke R^2^). The probability of being infected with *Blastocystis* sp. was similar for individuals from both Spain and the rest of Europe, but significantly increased for individuals from America and especially from Africa. No significant association was detected for individuals from Asia, which may be due to their scarcity. Abdominal pain, anorexia and type 2 diabetes were significantly associated with *Blastocystis* sp. detection, but corticosteroid treatment was associated with the absence of this microorganism. Infection was strongly associated with the presence of pathogenic bacteria and halitosis; namely, individuals with bacterial co-infection were 132.960 times more likely to be infected with *Blastocystis* sp. and this likelihood increased to 315.220 times more when halitosis was detected. However, the strongest association corresponded to relative eosinophilia; as relative eosinophilia increased by 1 unit, the likelihood for *Blastocystis* sp. infection increased 1105.260 times. The accuracy parameters for the binomial logistic regression model were high and were as follows: Sn = 0.967 (95% CI: 0.969–0.998); Sb = 0.998 (95% CI: 0.996–0.998); PPV = 0.983 (95% CI: 0.968–0.997); NPV = 0.997 (95% CI: 0.995–0.999). The area under the ROC curve was 0.997 (95% CI: 0.993–1.000; *p* > 0.001).

## 4. Discussion

*Blastocystis* sp. is among the most prevalent parasites found in human fecal specimens in diagnostic microbiology laboratories, although its clinical significance is still uncertain in contrast to common protozoa, which are responsible for a significant proportion of diarrheal morbidity globally, such as *Giardia duodenalis* and *Cryptosporidium* sp. [40,41,42]. In Spain, previous studies in patients that reported digestive disorders and attended medical services have shown that the percentage of patients infected with *Blastocystis* sp. is twice or even ten times higher than those infected with *G. duodenalis* and *Cryptosporidium* sp., respectively [28]; however, studies in asymptomatic children have reported similar infection rates for *Blastocystis* sp. and *G. duodenalis* [26].

The prevalence of *Blastocystis* sp. infection in patients with gastrointestinal symptoms in this study was 9.18%. This result is consistent with previous studies in central Spain using conventional microscopy, which have documented prevalence rates of 9.6% in HIV-positive children and 5.3–19.4% in children attending daycare centers and primary schools [24,25], while 13% was found in asymptomatic school children using PCR-based methods [26]. Higher prevalence rates have been reported in adult patients in Catalonia (northeastern Spain) through microscopic examination and PCR (27.8%) [27], and up to 35.2% of PCR-*Blastocystis* positive samples were found in humans sharing households with dogs and cats in northern Spain [43]. In Europe, the prevalence of this protist has been reported to range from 3% to 7% in France, Italy and the United Kingdom using direct-light microscopy, but higher rates (14.5−24.2%) were found using PCR-based methods in France, the Netherlands and Denmark [22,28,44,45,46,47].

It is significant to mention that the infection rates detected in this study may be an underestimation, since molecular analyses are known to be much more sensitive than microscopic techniques and xenic in vitro culture for the detection of *Blastocystis* sp. in fecal specimens from both humans and animals [1,45]. Culture from stool samples was significantly more sensitive than direct microscopic examination, but it is time consuming and not practical for diagnosis when a quick turnaround is required [45]. The sensitivity of microscopy has been suggested to increase when increasing the number of investigated samples. Microscopy on two SAF (sodium acetate-acetic acid-formalin) preserved samples in the test called “triple faeces test”, which combines multiple fecal sampling (on 3 consecutive days) with a concentration method, has been reported to provide a similar sensitivity to sequence confirmed-PCR [47,48]. In contrast, other studies have shown that microscopic diagnosis through a concentration technique did not increase when two or three consecutive stool samples were investigated, compared with one simple stool investigation [49].

Geographical location has been reported to influence the prevalence of parasitic infections [50]. In this study, a significant effect of geographic origin of patients on the prevalence of *Blastoscystis* sp. was found, with over 25% of infected patients originating from outside Europe, with the percentage of patients from Central and South America, and especially from Africa, being significantly higher in the cases than in controls. Poor sanitary and hygiene conditions have been proposed to explain the higher prevalence of *Blastocystis* sp. in low-income countries, since it is mainly transmitted through the fecal-oral route via consumption of contaminated food or water [3]. Infections rates ranging from 80.4% to 100% have been documented in school children in Senegal, 64% in Moroccan children, 71.1% in immigrant workers in Qatar, 78% in the Guinean population, and 63% in children in Lebanon [4,51,52,53,54,55]. In contrast, much lower rates are documented from European countries, including Denmark (5.6%), France (13.7–23.1%), Czech Republic (24%) and Spain (13%) [22,29,56,57].

In this study, the presence of *Blastocystis* sp. was not associated with either of the two age classes (children or adults) or with sex, although the cases were significantly younger than controls when age was considered as a quantitative variable. Many studies in different parts of the world have investigated the effect of host sex on *Blastocystis* sp. infection rates and most of them have shown that prevalence is not significantly related to sex [57,58,59], although some studies have shown a higher prevalence in either females [60] or males [61]. Similarly, there are contradictory results regarding the association with host age. Some studies found no significant relationship [21,59,62], but others reported higher *Blastocystis* sp. infection rates among younger adult age groups [22,23], adults aged more than 18 years [61] or even older than 60 years [63]; other studies have documented patients younger than 30 years or children in the group of age 5–15 years to have a higher risk of being infected with *Blastocystis* sp. [43,55].

Our results for BMI showed a highly significant difference between the groups of patients, with underweight individuals being significantly more common among the cases than in controls. This finding is consistent with previous studies that claimed a positive correlation between low BMI and *Blastocystis* sp. infection, an observation which has been related to a potential negative effect of the protist on dietary intakes and energy metabolism [42,64,65]. This potential negative impact on host weight could be of relevance when considering data that suggest that *Blastocystis* sp. is capable of long-term host colonization, with some individuals testing positive for up to 10 years [12].

Another potential factor that determines the transmission of pathogens is host immunity, since it is well known that patients with decreased immunity are particularly susceptible to opportunistic infections [66]. *Blastocystis* sp. has been widely reported in both immunocompetent and immunocompromised individuals but its pathogenic, or rather opportunistic, role has yet not been clearly elucidated. Recent systematic reviews and meta-analyses have estimated that the global pooled prevalence rate of this protist in immunocompromised patients is around 10% [67], and case–control studies have shown that immunosuppressive conditions, such as cancer, organ transplantation and hemodialysis, were associated with higher odds of infection [68]. In contrast, similar or even lower prevalence was reported in some studies on immunodeficient patients compared to controls [23]. In this study, immunosuppressive treatments with chemotherapy, monoclonal antibodies or antiretroviral drugs were more common in cases than in controls, which could support the role of *Blastocystis* sp. as an opportunistic organism. However, it is worth mentioning that treatment with corticosteroids was much more frequent in controls than in cases (28.9% compared to 0.9%), and the use of these immunomodulatory drugs was also significant as a potential protective factor in the model for binary logistic regression. The reason for this observation is not known; in fact, a recent study on patients with inflammatory bowel disease showed no clear association between the presence of *Blastocystis* sp. or the use of corticosteroids [69].

In this study, type 2 diabetes was significantly more frequent in cases and this metabolic disease was also a significant factor in the binary logistic regression. Most studies have also reported patients with diabetes mellitus to be at higher risk of infection, which has been related to a weakened immune system [70]. The presence of diabetes mellitus increased the risk of *Blastocystis* sp. infection more than 9 times in patients with irritable bowel syndrome in Egypt [71], although previous studies in Spain found no correlation among these factors [23]. In contrast, our results do not support *Blastocystis* infection to be a risk factor for irritable bowel syndrome, which was even more common among controls than in cases. The implication of *Blastocystis* sp. in the development of IBS is controversial, although some studies have indicated a positive association and suggested that accurate diagnosis of this protist should be included in the clinical protocol of IBS patients [72].

The odds ratio for halitosis, pathogenic bacteria and relative eosinophilia shows very high values. When both the odds of achieving an outcome, if exposed (presence of *Blastocystis,* cases) and if not exposed (absence of *Blastocystis,* controls), are very low, but odds in controls are even lower than odds in cases, high values for OR are found. However, 95% confidence intervals are very wide, pointing to a low confidence in the obtained OR values. Therefore, these high OR values found for halitosis, pathogenic bacteria and relative eosinophilia suggest than these factors are associated with *Blastocystosis,* but one must take into account that these OR values may be overestimated, due to the low frequencies of these outcomes in the whole sample of cases and controls.

Relative eosinophilia has been associated with *Blastocystis* sp. infection in this study. In parasitic diseases, blood eosinophilia is usually associated with helminth infections, especially coinciding with the larval migration through tissues, and travelers/immigrants from resource-limited countries, who are most likely to acquire these infections, have been reported to have a high likelihood of eosinophilia. However, it is usually considered uncommon for an eosinophilia to be produced by protozoan infections [73]. Few studies have reported a high proportion of eosinophils in the peripheral blood of symptomatic patients infected with *Blastocystis* sp. [74], but our results indicate that patients with relative eosinophilia are more likely to be infected, suggesting that eosinophilia should be taken into consideration in the diagnosis of *Blastocystis* sp. infection.

Human gut microbiota composition is considered a deciding agent in the pathogenicity and occurrence of *Blastocystis* sp., and the alteration of the intestinal environment provoked by pathogens has been suggested to be involved in its pathogenicity [15]. In healthy children, Kodio et al. (2019) [75] concluded that *Blastocystis* sp. colonization is associated with a higher diversity of the bacterial communities in the gut but is not associated with the presence of potentially pathogenic bacteria in the human gut. As higher bacterial diversity is commonly associated with health and lower incidence of inflammatory diseases, it has been suggested that *Blastocystis* colonization is associated with a healthy gut microbiome [76]. In this study, the cluster analysis showed that this protist was by far the most frequently detected intestinal parasite, and most patients infected had no presence of other helminths or protozoa included in the principal component analysis; however, co-infections with pathogenic bacteria or enteric parasites were much more common among cases than controls, which suggest that they are associated with *Blastocystis* sp. presence. These findings contrast with the observations of Hidalgo et al., (2019) [23] who suggested that the presence of other parasites significantly decreased the risk of positive detection of *Blastocystis* sp., although the authors indicated that there could be evidence of bias in the study because all the participants were patients submitted for parasitological diagnosis.

Whether *Blastocystis* sp. is a pathogen or a commensal of the human gut is still uncertain. Studies on healthy, randomly sampled individuals have shown a high presence of *Blastocystis* sp. and a prolonged colonization of the gut, but others have implicated it in intestinal diseases, including inflammatory bowel disease (IBD), irritable bowel syndrome (IBS) and even cutaneous disorders. The most common intestinal symptoms attributed to *Blastocystis* infection are diarrhea and abdominal pain, as well as nonspecific symptoms, such as nausea, vomiting, fatigue and flatulence [19,22,64,67]. The findings of the current study support a potentially pathogenic role for this protist, since most symptoms reported by infected patients were significantly more common in cases than in controls, including nausea, aerophagia, urticaria, anal itching and dyspepsia; moreover, the likelihood of having a *Blastocystis* infection increased with abdominal pain, anorexia and halitosis. In contrast, a higher proportion of patients in the control group presented episodes of diarrhea and fever, with the latter being even significantly more common in controls than in cases. However, our National Health Service only analyzes samples from patients attending consultations because of the presence of symptoms. For this reason, no controls without disease manifestations were available. Therefore, no conclusions on the pathogenic potential of *Blastocystis* could be directly reached from the analysis of our data set.

Diagnosis based only by the microscopic examination of stool specimens, lacking information relating to subtypes, is also a limitation of this study. Remarkable genetic diversity has been revealed among *Blastocystis* sp. from humans and animals, and differences in clinical significance have been suggested for different subtypes, an aspect which was not investigated in this study; however, at present, there is no widely accepted distinction between the pathogenic and non-pathogenic subtypes [8,16].

## 5. Conclusions

In summary, the results of the present study demonstrate a high prevalence of *Blastocystis* sp. infections in individuals with gastrointestinal illness in this geographical area, with most infected patients testing negative for protozoa or helminths. The probability of being infected with *Blastocystis* sp. significantly increased for underweight individuals and those native from Central and South America, and especially from Africa. Relative eosinophilia and some gastrointestinal symptoms, especially abdominal pain, anorexia and halitosis, but not diarrhea or fever, were associated with *Blastocystis* sp. detection. Co-infections with pathogenic bacteria were also related with *Blastocystis* sp. presence. A higher proportion of *Blastocystis* sp.-infected patients had type 2 diabetes and received some immunosuppressive treatments, such as chemotherapy, monoclonal antibodies or antiretroviral drugs, which could support the role of *Blastocystis* sp. as an opportunistic organism. This is the first case–control study of *Blastocystis* sp. in humans in northern Spain. Further research using molecular-based methods is needed to investigate the level of molecular diversity of *Blastocystis* sp. in this geographic area, and to ascertain the link between subtypes and factors associated with a higher risk of infection.

## Figures and Tables

**Table 1:** Distribution of demographic/anthropometric parameters and immunosuppressive treatment in cases (*Blastocystis*-positive patients) and in controls (*Blastocystis*-negative patients).

Variable	*Blastocystis*-Positive Patients	*Blastocystis*-Negative Patients	*p*-Value
**Geographical origin**			<0.001
Spain	215/337 (63.8%) ^a^	2712/3343 (81.1%) ^b^	
Rest of Europe	33/337 (9.8%) ^a^	267/3343 (8.0%) ^a^	
Africa	48/337 (14.20%) ^a^	165/3343 (4.9%) ^b^	
America	30/337 (8.9%) ^a^	144/3343 (4.3%) ^b^	
Asia	11/337 (3.3%) ^a^	55/3343 (1.6%) ^b^	
**Age groups**			0.140
≤16 years	148/338 (43.8)	1321/3344 (39.5%)	
>16 years	190/338 (56.2%)	2023/3344 (60.5%)	
Sex			0.125
Male	180/338 (53.3%)	1629/3344 (48.7%)	
Female	158/338 (46.7%)	1715/3344 (51.3%)	
**Categorized BMI**			<0.001
Underweight (<18.4)	95/276 (34.4%) ^a^	266/2349 (11.3%) ^b^	
Normal (18.5–24.9)	92/276 (33.3%) ^a^	939/2349 (40%) ^b^	
Overweight (25–29.9)	55/276 (19.9%) ^a^	891/2349 (37.9%) ^b^	
Obesity 30–> 40	34/276 (12.3%) ^a^	253/2349 (10.8%) ^a^	
**Immunosuppressive treatment**			
Chemotherapy	6/337 (1.8%)	1/3344 (0%)	<0.001
Monoclonal antibodies	3/337 (0.9%)	0/3344 (0%)	0.001
Antiretroviral drugs	3/337 (0.9%)	0/3344 (0%)	0.001
Nucleoside analogs	1/337 (0.3%)	47/3344 (1.4%)	0.124
Corticosteroids	3/337 (0.9%)	965/3344 (28.9%)	<0.001
Age (years) *	30.18 ± 1.294	36.11 ± 0.511	<0.001
BMI (Kg/m^2^) *	21.21 (9.51)	24.89 (5.10)	<0.001

* Quantitative variable. Data are reported as counts (%), except for age (years), which is described as the mean ± SE, and for BMI (kg/m^2^), which is summarized as median and interquartile range (IQR). ^a,b^: Values within a row with different superscripts differ significantly at *p* < 0.050.

**Table 2:** Clinical symptoms, co-morbidities, co-infections, and hematological/biochemical parameters in cases (*Blastocystis*-positive patients) and in controls (*Blastocystis*-negative patients).

Variables	*Blastocystis*-Positive Patients	*Blastocystis*-Negative Patients	*p*-Value
**Clinical symptoms**			
Diarrhea	166/336 (49.4%)	1797/3344 (53.7%)	0.144
Abdominal pain	275/336 (81.8%)	975/3344 (29.2%)	<0.001
Nausea	36/336 (10.7%)	29/3344 (0.9%)	<0.001
Vomiting	47/336 (14%)	446/3344 (13.3%)	0.803
Constipation	24/336 (7.1%)	323/3344 (9.7%)	0.160
Anorexia	34/336 (10.1%)	27/3344 (0.8%)	<0.001
Fever	18/336 (5.4%)	972/3344 (29.1%)	<0.001
Aerophagia	39/336 (11.6%)	53/3344 (1.6%)	<0.001
Halitosis	5/336 (1.5%)	4/3344 (0.1%)	0.001
Urticaria	103/336 (30.7%)	202/3344 (6%)	<0.001
Anal itching	32/336 (9.5%)	90/3344 (2.7%)	<0.001
Dyspepsia	27/336 (8%)	88/3344 (2.6%)	<0.001
**Co-morbidities**			
Celiac disease	8/336 (2.4%)	48/3344 (1.4%)	0.264
Type 2 diabetes	59/309 (19.1%)	182/3344 (5.4%)	<0.001
IBS	6/336 (1.8%)	160/3344 (4.8%)	0.017
Cancer	18/336 (5.4%)	194/3344 (5.8%)	0.833
**Co-infections**			
Pathogenic bacteria	30/336 (8.9%)	6/3344 (0.2%)	<0.001
Helminths	22/336 (6.5%)	22/3344 (0.7%)	<0.001
Protozoa	25/336 (7.4%)	72/3344 (2.2%)	<0.001
**Laboratory parameters**			
Glucose *	87.00 (14.0)	87.00 (14.0)	0.379
Glycosylated hemoglobin *	5.70 (1.0)	5.50 (0.9)	0.523
Hemoglobin *	14.10 (1.80)	14.10 (2.50)	0.903
Relative eosinophilia *	3.20 (4.5)	0.10 (0.1)	<0.001

* Quantitative variable. Data are reported as counts (%) except for quantitative variables, which are described as median and interquartile range (IQR).

**Table 3:** Rotated component matrix: loadings of principal components 1, 2 and 3 on variables related to the absence/presence of pathogens. Coefficients below 0.3 (absolute value) have been suppressed for easier interpretation.

Parasite	Principal Component		
	1	2	3
*Isospora belli*	0.710		
*Strongyloides stercoralis*	0.601	−0.433	
*Giardia lamblia*	0.594		
*Dientamoeba* sp.	0.429		
*Entamoeba* sp.		0.647	
*Ascaris lumbricoides*		0.619	
*Enterobius vermicularis*			0.801
*Blastocystis* sp.		0.308	0.607

**Table 4:** Characteristics of individuals included in the 6 clusters obtained in the study. Absence or presence of a particular pathogen was recorded as 0 and 1, respectively.

**Cluster**	**Frequency**	***Ascaris lumbricoides***		***Dientamoeba* sp.**		***Strongyloides stercoralis***		***Entamoeba* sp.**		***Enterobius vermicularis***		***Gardia lamblia***		***Isospora belli***		***Blastocystis* sp.**	
		**0**	**1**	**0**	**1**	**0**	**1**	**0**	**1**	**0**	**1**	**0**	**1**	**0**	**1**	**0**	**1**
1	3277/3680 (89%)	3277		3277		3277		3277		3277		3277		3277		3277	
2	53/3680 (1.4%)	53		45	8	53		53		53		8	45	53		44	9
3	16/3680 (0.4%)	16		16		16		16			16	16		16		8	8
4	23/3680 (0.6%)	18	5	22	1	23		4	19	23		21	2	22	1	11	12
5	10/3680 (0.3%)	10		9	1	4	6	10		10		5	5	5	5	4	6
6	301/3680 (8.2%)	301		301		301		301		301		301		301			301
Total	3680	3675	5	3670	10	3674	6	3661	19	3664	16	3628	52	3674	6	3344	336

**Table 5:** Description of the number of patients with Blastocystosis and co-infections included in each cluster, except cluster 1 (only controls) and cluster 6 (patients carrying exclusively *Blastocystis*).

Cluster Number	Patients Infected with *Blastocysts* sp.	Patients with Co-Infection	Co-Infection
2	9	4/336 (1.2%)	*Giardia lamblia*
		5/336 (1.5%)	*Dientamoeba* sp.
3	8	8/336 (2.4%)	*E. vermicularis*
4	12	6/336 (1.8%)	*Entamoeba* sp.
		1/336 (0.3%)	*Entamoeba* sp./*G. lamblia*
		4/336 (1.2%)	*A. lumbricoides*
		1/336 (0.3%)	*A. lumbricoides/Dientamoeba* sp./*Entamoeba* sp./*G. lamblia*/*I. belli*
5	6	4/336 (1.2%)	*G. lamblia*/*I. belli*
		1/336 (0.3%)	*S. stercoralis/I. belli*
		1/336 (0.3%)	*Dientamoeba* sp./*S stercoralis*/*I. belli*

**Table 6:** Final model for binary logistic regression, showing the significant associations of blastocystosis to patient’s origin, presence of pathogenic bacteria, abdominal pain, anorexia, halitosis, type 2 diabetes and corticosteroid treatment, and relative eosinophilia (quantitative variable).

Variable	B	SE	Wald	df	*p*-Value	Odds Ratio (OR)	95% CI for Odds Ratio	
							Lower	Upper
Geographical origin			9.205	4	0.056			
Rest of Europe	−0.141	0.918	0.024	1	0.878	0.868	0.144	5.250
Africa	2.025	0.810	6.249	1	0.012	7.574	1.548	37.046
America	1.828	0.873	4.391	1	0.036	6.223	1.125	34.411
Asia	0.726	1.629	0.199	1	0.656	2.067	0.085	50.360
Pathogenic bacteria	4.890	1.367	12.804	1	<0.001	132.960	9.130	1936.310
Abdominal pain	2.568	0.576	19.901	1	<0.001	13.041	4.220	40.300
Anorexia	3.135	0.943	11.055	1	0.001	22.999	3.623	146.016
Halitosis	5.753	1.632	12.433	1	<0.001	315.220	12.875	7717.767
Type 2 diabetes	2.021	0.722	7.837	1	0.005	7.547	1.833	31.070
Corticosteroids treatment	−5.998	1.574	14.514	1	<0.001	0.002	0.000	0.054
Relative eosinophilia	7.008	0.658	113.374	1	<0.001	1105.260	304.259	4014.997
Constant	−9.487	0.831	130.250	1	<0.001	0.000		

B: regression coefficient; SE: standard error; df: degrees of freedom; 95% CI: 95% interval of confidence.

## Data Availability

Availability of data will depend on specific permission from the Ethics Committee of Aragón.
